# Simulation of Diffusion-Controlled Growth of Interdependent Nuclei under Potentiostatic Conditions

**DOI:** 10.3390/ma15103603

**Published:** 2022-05-18

**Authors:** Alexander V. Kosov, Olga V. Grishenkova, Vladimir A. Isaev, Yuriy Zaikov

**Affiliations:** Institute of High Temperature Electrochemistry, Ural Branch of the Russian Academy of Sciences, 620990 Yekaterinburg, Russia or olagris@mail.ru (O.V.G.); v.isaev@ihte.uran.ru (V.A.I.); zaikov@ihte.uran.ru (Y.Z.)

**Keywords:** electrocrystallization, computer simulation, Voronoi tessellation, diffusion, kinetics

## Abstract

The problem of diffusion-controlled growth following an instantaneous nucleation event was studied within the framework of a new numerical model, considering the spatial distribution of hemispherical nuclei on the electrode surface and the mutual influence of growing nuclei via the collision of 3D diffusion fields. The simulation of the diffusion-controlled growth of hexagonal and random ensembles was performed at the overpotential-dependent number density of nuclei. The diffusion flow to each nucleus within a random ensemble was simulated by the finite difference method using the derived analytical expressions for the surface areas and the volumes formed at the intersection of 3D diffusion fields with the side faces of a virtual right prism with a Voronoi polygon base. The implementation of this approach provides an accurate calculation of concentration profiles, time dependences of the size of nuclei, and current transients. The results, including total current density transients, growth exponents, and nucleus size distribution, were compared with models developed within the concept of planar diffusion zones, the mean-field approximation and the Brownian dynamics simulation method, as well as with experimental data from the literature. The prospects of the model for studying the initial stages of electrocrystallization were discussed.

## 1. Introduction

The initial stages of electrocrystallization are one of the key moments, both for fundamental studies of phase formation and for the development of electrochemical nanotechnologies [1,2,3]. The simplest way to obtain data on the mechanism and kinetics of the process is to study the nucleation/growth regularities at constant supersaturation (overpotential), i.e., under potentiostatic conditions. However, an exact mathematical description of the diffusion-controlled growth of new-phase nuclei formed on the electrode surface remains an insoluble problem, even in this case. The main difficulty arises from the development of diffusion fields around growing nuclei, which affect the nucleation and growth rates in their vicinity [4].

Analytical expressions for potentiostatic current transients can be derived only using certain assumptions, e.g., within the framework of the so-called “concept of planar diffusion zones” [5,6,7,8,9,10,11,12]. In this approach, the gradual overlap of 3D diffusion fields of growing hemispherical nuclei is replaced by the overlap of 2D equivalent diffusion zones on the electrode surface, and this problem is solved using Kolmogorov–Johnson–Mehl–Avrami (KJMA) theory [13,14,15]. Thus, the current density is proportional to $\theta =1-\exp(-\theta_{\mathrm{ex}})$, where *θ* and *θ*_ex_ are the fractions of the area covered by diffusion zones with and without considering the overlap, respectively. The radius of each zone is determined from the condition that the amount of material diffusing towards it by linear diffusion is equal to that transferred to the hemispherical growing center through spherical diffusion. The nuances associated with the zone growth rate and the nucleation rate led to several solutions for progressive nucleation. Equations for the limiting case of instantaneous nucleation (site-saturation nucleation), when nuclei are formed at all active centers of the electrode surface at *t* = 0, were also given in [5,6]:

(1) $$i=zec_{0}{\left(\frac{D}{\pi t}\right)}^{1/2}\left[1-\exp\left(-\pi \tilde{k}DN_{0}t\right)\right],$$

(2) $${\left(\frac{i}{i_{m}}\right)}^{2}=\frac{1.9542}{t/t_{m}}{\left\{1-\exp\left[-1.2564\left(t/t_{m}\right)\right]\right\}}^{2},$$

(3) $$i_{m}=0.6382zec_{0}D{\left(\tilde{k}N_{0}\right)}^{1/2},\;t_{m}=1.2564/\pi \tilde{k}DN_{0},$$

where *i* (A⋅cm^−2^) is the current density, *z* is the valency of depositing ions, *e* (C) is the elementary electric charge, *c*_0_ (cm^−3^) is the bulk concentration of depositing ions, *D* (cm^2^ c^−1^) is diffusion coefficient, *t* (s) is time, $\tilde{k}={\left(8\pi c_{0}\upsilon \right)}^{1/2}$, υ (cm^3^) is the volume of one atom of the deposit, *N*_0_ (cm^−2^) is the number density of sites, and *i*_m_ and *t*_m_ are the coordinates of the maximum. Despite significant approximations, the above models provide a qualitatively correct prediction of the shape of the experimental current transients. In addition, the Scharifker–Hills (SH) model [5,6] describes adequately the current at θ → θ_ex_ and θ → 1, i.e., spherical diffusion to independent nuclei and planar diffusion to the electrode, respectively. The Scharifker–Mostany (SM) model [7] gives underestimated values of the current up to the transition to the Cottrellian behavior [12,16,17]. Sufficiently simple procedures for extracting quantitative information about the nucleation and growth parameters from experimental current transients using models [6,7] determine their wide practical application. However, the analysis based on dimensionless dependences [6] is criticized for masking the actual discrepancies between the experimental and theoretical curves [11]. Moreover, values of the number density of active sites and/or the nucleation rate constant calculated from the maximum coordinates [6] or by fitting [7] often differ by several orders of magnitude from the values found using microscopic (SEM, AFM, TEM, etc.) observations [18,19,20,21].

The validity of this approach and the reliability of the results obtained using the above models are still being discussed. Abyaneh believes [22] that Equation (1) and other equations of current transients in models [4,5,6,7,8,9,10] describe the growth of right-circular cones, and not of hemispheres, since they “fail to mathematically address the effects of the overlap of diffusion zones on the overall rate of electrocrystallisation”. On the contrary, according to Politi and Tomellini [23], the “planar diffusion zones” approach actually provides a good approximate solution to the complex problem of diffusion-controlled growth due to the interference between diffusion fields, at least for simultaneous nucleation. Moreover, the application of this approximation to modify the stochastic approach, which considers the processes of 3D nucleation and growth without the interaction of diffusion fields [23,24,25], is useful for determining the nucleus growth law, mean film thickness, and surface coverage of the electrode [23].

More complex approaches were proposed in [26,27,28,29,30,31] to describe the kinetics of diffusion-controlled growth following instantaneous nucleation within the framework of various approximations. Di Biagio and Tomellini [26] studied the effect of the correlation between diffusion fields on the growth of semicircular nuclei by numerical integration of the diffusion equation by the finite difference method, adapted the Voronoi cell method [32] and the linear diffusion approximation for modeling the kinetics of 3D electrodeposition, and explained the change in the growth rate of hemispherical nuclei. Tomellini and Fanfoni [27,28] considered the relationship between the KJMA kinetics and Voronoi diagrams.

Bobbert [29] solved the problem of diffusion to an ensemble of slowly growing hemispherical nuclei on a substrate using the mean field approximation: the nuclei were considered to be point sinks and the inhomogeneous diffusion equation was solved by means of the Green function. In numerical simulation, the random spatial distribution of nuclei was additionally taken into account and a correction was introduced for microscopic fluctuations of the concentration field. As a result, the reduced current transients deviated from the SH model at *t*/τ > 2.5, where $\tau =\pi \tilde{k}DN_{0}$, and dispersion in the sizes of the nuclei was about 5%. Tokuyama [30,31] used a similar approach and showed that the static many-body (screening) effect leads to a change in the growth exponent for the average nucleus radius from 1/2 to 1/6, the dynamic many-body (correlation) effect causes dispersion of the average size of the nuclei, and the behavior of the average current transient depends on the volume fraction of the nuclei.

Cao et al. [33] performed direct numerical simulations using the boundary integral method to study instantaneous and progressive nucleation followed by diffusion-controlled growth. The Monte Carlo method was used to simulate nucleation, and a Green’s function based on spherical sources was used to calculate the concentration of depositing ions at any point in space and time. The application of this method was limited to the case when the distance between nuclei is 5–10 times greater than the radius of the nucleus. For instantaneous nucleation, dimensionless transients *i*/*i*_m SH_ vs. *t*/*t*_m SH_ (*i*_m SH_ and *t*_m SH_ were calculated from Equation (3)) were identical to those obtained in [29,30,31].

Nagy et al. [34,35] proposed a model of diffusion-controlled electrodeposition process using a 3D random walk algorithm. During simulation, the authors determined the current associated with the growth of one hemispherical nucleus placed at the bottom of a prism with a square base. The effect of neighboring domains was taken into account when the diffusion layer reached the side walls of the simulation box [35]. At a low concentration of depositing ions, the simulated current transients for nuclei located on a square lattice were close to the SH model.

Fransaer and Penner [36,37] applied the Brownian dynamics simulation method to study the diffusion-controlled growth of random and hexagonal ensembles of nanosized silver hemispheres following an instantaneous nucleation event on the surface of a graphite end electrode during the electrodeposition from solution. For a random ensemble, a qualitatively good agreement was found with the dependences calculated by Equation (1). The main distinction was the lower *i*_m_ value compared to the SH model (especially at large *N*_0_). The difference in the size of nuclei increased as the electrodeposition time increased and the distance to the nearest neighbor decreased. For hexagonal ensembles, noticeable deviations of *i*(*t*) from the theoretical dependences [38,39] and an insignificant size dispersion were observed.

Numerical simulation is certainly a powerful tool for the quantitative description of complex processes [40,41,42,43,44,45], including nucleation and diffusion-controlled growth of randomly distributed nuclei. However, simulating the diffusion process as collisions among multiple random walkers using the standard kinetic Monte Carlo method (KMC) is associated with serious computational limitations, when the concentration of diffusing particles is low. To address this issue, Oppelstrup et al. [42] proposed an algorithm based on the theory of first-passage processes and time dependent Green’s functions to propagate the walkers over long distances in a single KMC step. The finite difference method is another suitable option. For example, Zargarnezhad and Dolati [43] applied a finite-difference method of 3D Crank-Nicholson scheme over non-uniform grid distribution with an adaptive time-step in the vertical direction to a substrate to simulate mass transfer in solution. Pricer et al. [44] and Saedi [45] successfully used a 1D finite difference model for the mass transfer of electroactive species in the electrolyte.

In this work, we propose an approach for numerical simulation of the diffusion-controlled growth following an instantaneous nucleation event. We use the usual approximations of the classical theory of nucleation and the finite difference method to simulate the fluxes of depositing ions to each hemispherical nucleus growing by the direct attachment mechanism under potentiostatic conditions. The results for random and hexagonal ensembles are compared with the SH model and literature data.

## 2. Model and Calculation Method

### 2.1. Hexagonal Ensemble

**Figure 1:**
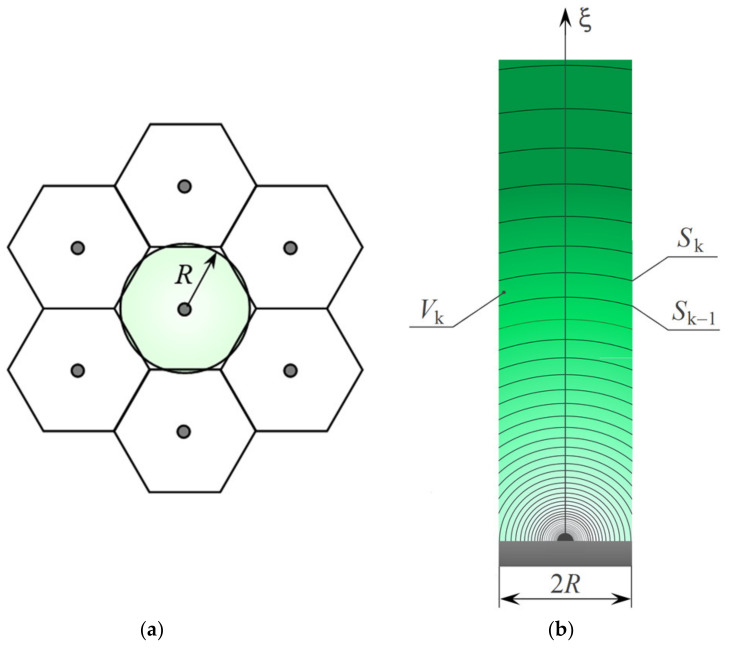
(**a**) Part of a hexagonal ensemble (a top view); (**b**) Cross section of the virtual diffusion cylinder of the nucleus equidistant from six similar neighbors. The scheme illustrates the change in the concentration of depositing ions (via a color gradient) and the mass transfer conditions inside a virtual cylinder with a base radius *R* due to the growth of a nucleus within the ensemble.

In the case of a uniform distribution of identical nuclei over the electrode surface, mass transfer to each nucleus (extreme nuclei are not considered in this work) will occur through spherical diffusion inside a virtual right prism with a base in the form of a regular hexagon (Figure 1). A good approximation in this case is to replace the prism with a cylinder with base radius *R* (cm), provided that the base area of the cylinder is equal to the area of this hexagon.

When the number density of nuclei is low and/or the electrodeposition time is short, the nuclei will not affect each other’s growth. Then the radius of any nucleus, *r* (cm), and the growth current, *I*_g_ (A), can be easily found using the well-known expressions [46,47,48]:

(4) $$r={\left(2D\upsilon c_{0}\right)}^{1/2}{\left[1-\exp(-f\eta )\right]}^{1/2}t^{1/2},$$

(5) $$I_{g}=\pi ze\upsilon^{1/2}{\left(2Dc_{0}\right)}^{3/2}{\left[1-\exp(-f\eta )\right]}^{3/2}t^{1/2},$$

where $f=ze/k_{B}T$, *k*_B_ (J K^−1^) is the Boltzmann constant, *T* (K) is the absolute temperature, and *η* (V) is the overpotential.

Otherwise, the growth rate of nuclei will gradually decrease due to the collision of their 3D diffusion fields. To solve this problem, let us consider the growth of one nucleus equidistant from six nearest neighbors that affect the kinetics of its growth (Figure 1a). To simulate the mass transfer to the nucleus, we divide the volume of the virtual cylinder into *k* small volumes bounded from above and below by hemispherical surfaces or surfaces formed by the intersection of cylindrical walls with hemispheres of different radius (Figure 1b). The radii, *R*_k_ (cm), surface areas, *S*_k_ (cm^2^), and volumes, *V*_k_ (cm^3^), can be found as follows:

(6) $$R_{k}=\gamma^{k}r,$$

(7) $$S_{k}=\left\{\begin{array}{l} 2\pi R_{k}^{2},\;\xi <R\\ 2\pi R_{k}[R_{k}-{\left(R_{k}^{2}-R^{2}\right)}^{1/2}]\;,\;\xi \geq R \end{array}\right.,$$

(8) $$V_{k}=\left\{\begin{array}{l} 2\pi R_{k}^{2}/3,\;\xi <R\\ 2\pi \;[R_{k+1}^{3}-R_{k}^{3}-{\left(R_{k+1}^{2}-R^{2}\right)}^{3/2}+{\left(R_{k}^{2}-R^{2}\right)}^{3/2}]/3,\;\xi \geq R \end{array}\right.,$$

where k = 1, 2, etc., ξ (cm) is the distance from the center of the nucleus, and γ is a coefficient whose value is slightly greater than 1. The growth of the nucleus leads to a gradual change in the concentration of depositing ions in each of the *V*_k_ volumes. The change in the concentration profile *c*(ξ,*t*) for a small interval, Δ*t* (s), is associated with a change in the number of depositing ions, Δ*n*_k_, in each small volume due to flows through the upper and lower boundaries (i.e., the influx from *V*_k+1_ and the outflow in *V*_k–1_). Then

(9) $$\Delta c_{k}=c_{k}(t+\Delta t)-c_{k}(t)=(\Delta n_{k+1}-\Delta n_{k})/V_{k},$$

(10) $$\Delta n_{k}=-DS_{k}(c_{k}-c_{k-1})\Delta t/\Delta \xi ,\;k>1,$$

where Δξ (cm) is the distance between the centers of adjacent volumes. Equation (10) was obtained using the equations of Fick’s first law and the particle flux density, j=Δn/SΔt. For the volume *V*_1_ adjacent to the nucleus of radius *r*, we have:

(11) $$\Delta n_{1}=-2\pi r^{2}j_{1}\Delta t,$$

(12) $$j_{1}=-D{\left(\partial c/\partial \xi \right)}_{\xi =r}.$$

This approach is suitable for calculating the current density spent on the growth of the nucleus,

(13) $$i_{g}=zeD{\left(\partial c/\partial \xi \right)}_{\xi =r},$$

and the time dependence of nucleus radius (taking into account the mass balance on the electrolyte/nucleus interface):

(14) $$\frac{d\;r}{d\;t}=\frac{i_{g}\upsilon }{ze}.$$

Finally, the total current for the hexagonal ensemble is found as

(15) $$I=2\pi r^{2}i_{g}N_{0}$$

because the growth current densities and sizes of all nuclei are the same in this case.

### 2.2. Random Distribution of Nuclei

**Figure 2:**
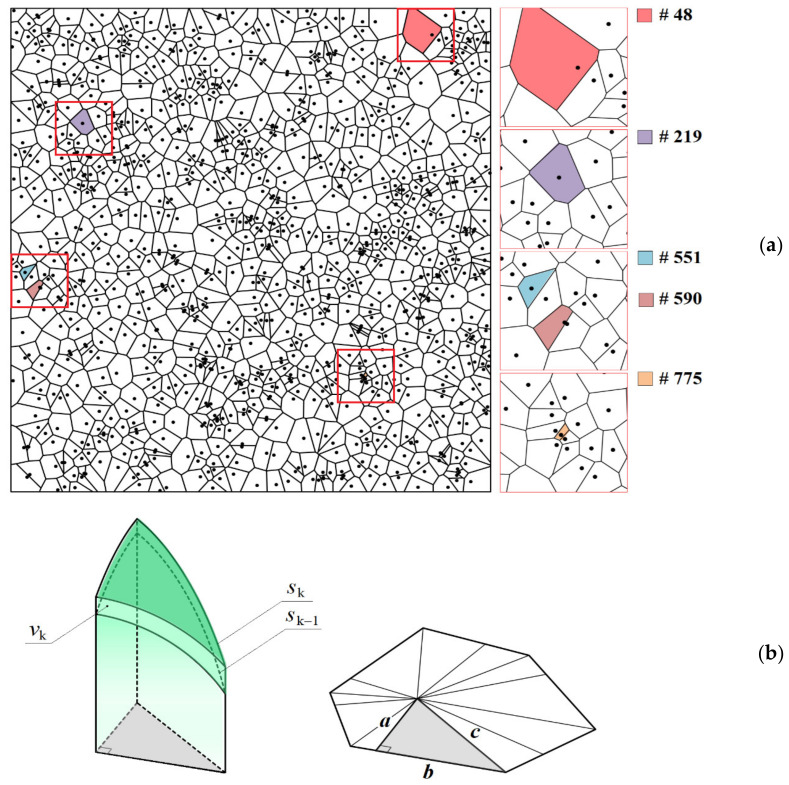
(**a**) Voronoi tessellation for 1000 nuclei and magnified images of selected domains. Areas of colored cells in ascending order of cell numbers (#), *S*_v_ (cm^2^): 6.13 × 10^−6^, 2.13 × 10^−6^, 7.06 × 10^−7^, 8.77 × 10^−7^, 1.17 × 10^−7^. Half-distance from nucleus to the nearest neighbor for the same cells, *R*_v_ (cm): 3.34 × 10^−4^, 6.19 × 10^−4^, 2.95 × 10^−4^, 4.89 × 10^−5^, 9.23 × 10^−5^. The results of the calculation using (**a**) and their discussion are given in Section 3.2. (**b**) Schematic illustration explaining how *S*_k_ and *V*_k_ are calculated at a random spatial distribution of nuclei. Symbols *a*, *b* and *c* are included in Equations (16)–(19).

The same approach was applied, but Voronoi tessellation was used to determine the area of the electrode surface related to each nucleus (Figure 2a). Accordingly, the mass transfer to any nucleus occurs through spherical diffusion inside a virtual right prism with a Voronoi polygon as a base. To find *S*_k_ and *V*_k_ in the case of a random spatial distribution of nuclei, each polygon was divided into rectangular triangles (Figure 2b) and the surface areas, *s*_k_, and volumes, *v*_k_, formed at the intersection of hemispheres of different radii, *R*_k_, with the side faces of a triangular prism were initially determined. For *s*_k_ and *v*_k_, the following analytical expressions were obtained:

(16) $$s_{k}=\left\{\begin{array}{l} R_{k}^{2}\arctan(b/a),\;R_{k}\leq a\\ R_{k}^{2}\arctan(b/a)-\pi R_{k}^{2}\left[1-(a/R_{k})\right]/2,\;a<R_{k}\leq c\\ AR^{2},\;R_{k}>c \end{array}\right.,$$

(17) $$v_{k}=\left\{\begin{array}{l} R_{k}^{3}\arctan(b/a)/3,\;R_{k}\leq a\\ R_{k}^{3}\arctan(b/a)/3-\pi R_{k}^{3}\left[{\left(a/R_{k}\right)}^{3}-3a/R_{k}+2\right]/12,\;a<R_{k}\leq c\\ BR_{k}^{3}/2,\;R_{k}>c \end{array}\right.,$$

where *a*, *b*, and *c* are the adjacent leg, opposite leg, and hypotenuse of this right triangle, respectively,

(18) $$A=\arctan\left(\frac{a}{b}\frac{\sqrt{R_{k}^{2}-c^{2}}}{R_{k}}\right)-\arctan\left(\frac{a}{b}\right)+\frac{a}{R_{k}}\arcsin\left(\frac{b}{\sqrt{R_{k}^{2}-a^{2}}}\right)$$

(19) $$B=\frac{ab}{3}\frac{\sqrt{R_{k}^{2}-c^{2}}}{R_{k}^{3}}+\frac{2}{3}\left[\arctan\left(\frac{a}{b}\frac{\sqrt{R_{k}^{2}-c^{2}}}{R_{k}}\right)-\arctan\left(\frac{a}{b}\right)\right]+\frac{a}{3R_{k}}\left[3-{\left(\frac{a}{R_{k}}\right)}^{2}\right]\arcsin\left(\frac{b}{\sqrt{R_{k}^{2}-a^{2}}}\right)$$

Summing the corresponding *s*_k_ and *v*_k_ gives a set of *S*_k_ and *V*_k_ for each polygon. Similar formulas can be used to calculate the volumes and surface areas of colliding nuclei whose radius exceeded half the distance to the nearest neighbor during growth. The total current of randomly distributed nuclei can be obtained by summing the individual contributions of all *N* nuclei, taking into account their actual surface area, *s*_N_ (cm^2^), and growth current density:

(20) $$I=\sum_{N}s_{N}i_{g\;N}.$$

In other respects, the calculation principle for *c*(ξ,*t*), *i*_g_(*t*), and *r*(*t*) does not differ from that described in Section 2.1. The proposed approach gives concentration profiles, time dependences of the radius, volume, surface area, and growth current of each nucleus. Thus, complete and accurate information about the evolution of the entire ensemble of nuclei is provided without any averaging.

### 2.3. Parameters and Simulation Procedure

A series of potentiostatic current transients for hexagonal ensembles was calculated in the overpotential range from 10 to 140 mV. The values of the radius *R* and the number density of nuclei, *N*_0_ = 1/(π*R*^2^), used in the simulation are given in Table 1. The values of *N*_0_ agree in order of magnitude with data from the literature [10,49] and increase exponentially as the overpotential increases. The exponential dependence *N*_0_(η) was experimentally confirmed for instantaneous nucleation, e.g., in [50].

Voronoi tessellations for modeling random ensembles were obtained using the Fortune algorithm [51]. In this work, the results are presented only for the diagram in Figure 2a, because changing the tessellation did not actually affect the total current transients at *N* = 1000. At the same overpotential, the *N*_0_ value for a random spatial distribution was equal to that for a hexagonal ensemble.

The initial nucleus radius, *r*_0_ (cm), slightly exceeded the critical size, *r*_c_ (cm):

(21) $$r_{0}=r_{c}+\varepsilon ,\;r_{c}=2\sigma \upsilon /ze\eta_{0},$$

where σ (J⋅cm^−2^) is the surface tension of the electrolyte/nucleus interface and ε = 10^−9^ cm.

The concentration of depositing ions near the nucleus surface, *c*_sr_ (cm^−3^), depended on the overpotential [46]:

(22) $$c_{\mathrm{sr}}=c_{0}\exp(-f\eta )$$

The finite-difference code was implemented in Excel using the built-in VBA programming language [52] to simulate the diffusion-controlled growth of each nucleus. Semi-infinite diffusion was simulated in this work at a maximum ξ value equal to 1 cm. The time step, Δ*t* = [*r*(γ−1)]^2^/(3*D*), ensured the stability, acceptable rate and accuracy of calculations at γ = 1.1. The error of the current value at the maximum point was 0.5%.

The concentration profile near the nucleus was approximated by the equation

(23) $$c(\xi )=m/\xi \;+\;n\xi^{2}\;+\;p\xi \;+\;q$$

at each time step. The coefficients of Equation (23) were found by solving a system of four equations using the Gauss method, taking into account *c*_sr_ and concentrations in the centers of volumes *V*_1_, *V*_2_, and *V*_3_. Thus, the growth current density was calculated using Equation (13) with

(24) $${\left(\partial c/\partial \xi \right)}_{\xi =r}=-m/r^{2}+2nr+p.$$

When the nucleus size increased by 0.1%, the radii of all surfaces were recalculated in accordance with Equation (6), i.e., all surfaces moved up (except for the fixed last one). The penultimate surface was destroyed if its radius exceeded 0.95 cm.

The electrocrystallization parameter values were close to those for silver electrodeposition from the AgNO_3_-KNO_3_-NaNO_3_ melt (*c*_Ag+_ = 0.0166 M) [53]: *T* = 523 K, *z* = 1, υ = 1.7 × 10^−23^ cm^3^, σ = 1 × 10^−5^ J cm^−2^, *D* = 1 × 10^−5^ cm^2^ s^−1^, *c*_0_ = 1 × 10^19^ cm^−3^. It was shown experimentally [46,53] and theoretically [54] for this system that, in a wide range of overpotentials, the rate of the charge transfer stage affects the kinetics of the nucleus growth only at the very initial stage, when the nucleus size is close to the critical one. The contributions of side processes, including surface diffusion, are also negligible [53,55], so this system is the most suitable for studying diffusion-controlled growth.

## 3. Results and Discussion

### 3.1. Hexagonal Ensemble

**Figure 3:**
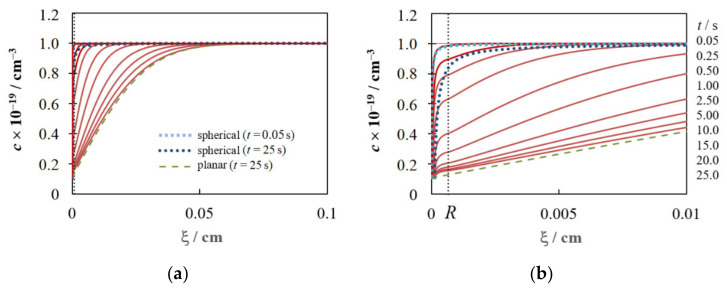
Concentration profiles for any nucleus within the hexagonal ensemble (dark red solid lines) for the ten *t* values indicated to the right of the figure (*t* = 0.05 s for the top profile and *t* = 25 s for the bottom one). Parameter values: *c*_0_ = 1 × 10^19^ cm^−3^, η = 100 mV and *R* = 6.49 × 10^−4^ cm (*N*_0_ = 7.55 × 10^5^ cm^−2^). For comparison, the concentration profiles for spherical diffusion to the independent nucleus (dotted lines) and planar diffusion to the electrode (a dashed line) are also shown here for the same *c*_0_ and η values and *t* = 0.05 s and/or *t* = 25 s; (**a**,**b**) differ only in ξ scale.

Typical concentration profiles for diffusion-controlled growth of a nucleus equidistant from six similar nuclei (see Figure 1a) are presented in Figure 3 at some time points in the range 0.05 s ≤ *t* ≤ 25 s (solid lines). For comparison, Figure 3 also shows the concentration profiles for stationary spherical diffusion to an independent nucleus [46], whose radius is described by Equation (4),

(25) $$c(\xi ,t)=c_{0}-(c_{0}-c_{\mathrm{sr}})\frac{r(t)}{\xi }$$

at *t* = 0.25 s and *t* = 25 s (dotted lines), and for planar diffusion to the electrode [56],

(26) $$c(\xi ,t)=c_{0}\;\mathrm{erf}\left[\frac{\xi }{2{\left(Dt\right)}^{1/2}}\right]$$

at *t* = 25 s (a dashed line).

Each concentration profile associated with mass transfer to a nucleus within a hexagonal ensemble can be characterized as follows: spherical diffusion to a nucleus always realized at ξ < *R*, and a gradual transition to planar diffusion to the electrode occurs as a result of propagation and collision of 3D diffusion fields of growing nuclei at ξ ≥ *R*. A slight inflection in the concentration profiles near ξ = *R* is a consequence of this transition. Mass transfer becomes purely planar at ξ > 10*R*. Figure 3 also demonstrates the change in the shape of the concentration profile as the electrodeposition time increases: the simulated profile practically coincides with that calculated by Equation (25) at small *t* (e.g., 0.05 s), and *c*(ξ,*t*) is close to that described by Equation (26) at large *t*. As far as we know, the concentration profiles for this case have not been calculated so carefully in other works.

**Figure 4:**
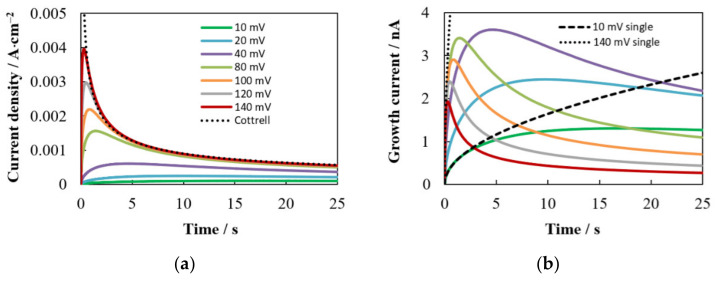

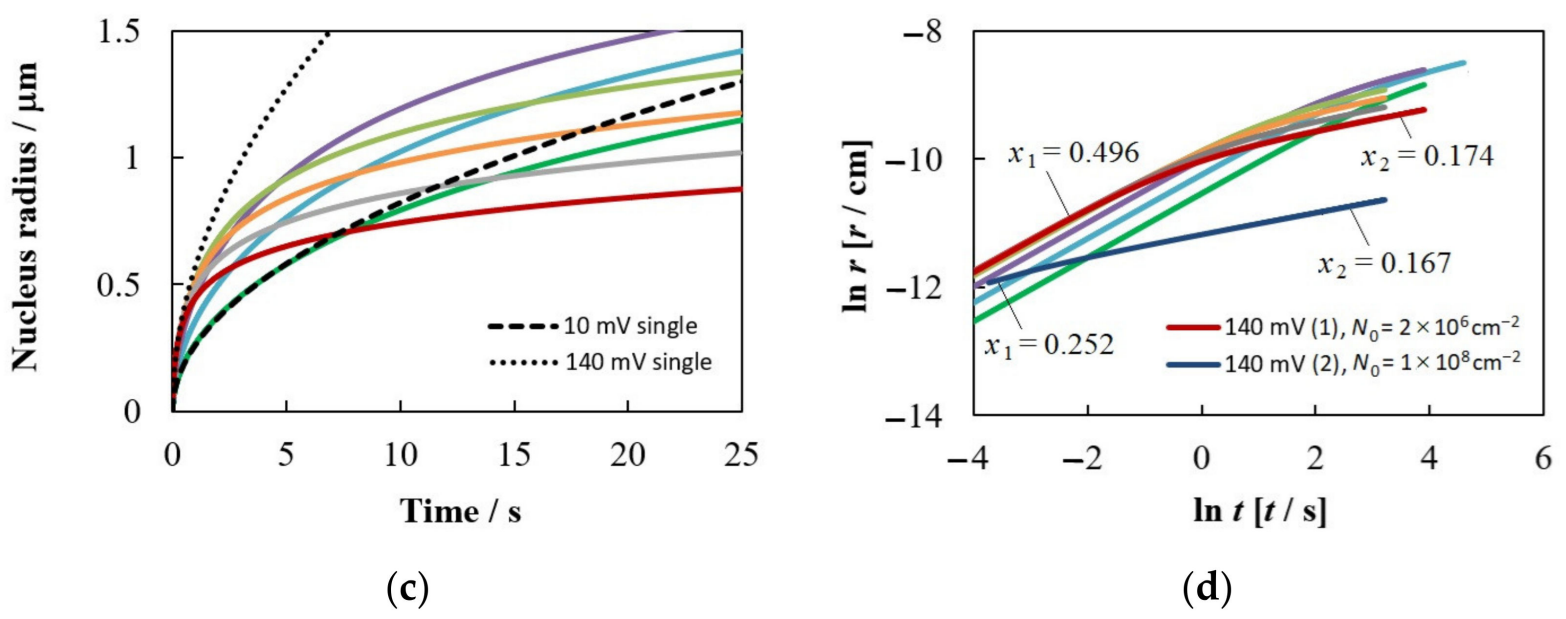
(**a**) Calculated potentiostatic current density transients, (**b**) time dependences of the growth current, (**c**) time dependences of the nucleus radius, and (**d**) logarithmic dependences of the nucleus radius on time for hexagonal ensembles. Designations of colored lines in (**b**,**c**) are given in (**a**). Parameter values are given in Section 2.3 and Table 1. The radius and growth current of a single nucleus were calculated using Equations (4) and (5), respectively. The cathode overpotential and current are considered positive in this work.

Figure 4a presents a series of potentiostatic current density transients for hexagonal ensembles in which the number density of nuclei increases and the distance between nearest neighbors decreases, according to Table 1, as the overpotential increases. It can be seen that the shape of the calculated *i*(*t*) dependences and their mutual arrangement are similar to those observed experimentally for many systems at a low concentration of depositing ions. Namely, as the overpotential increases, the maximum value of the current density (*i*_m_) increases, the time to reach the maximum (*t*_m_) decreases, and the final *i*(*t*) sections converge and merge with the curve described by the Cottrell equation.

Figure 4b,c show the time dependences of the growth current and nucleus radius, respectively. It can be seen that *I*_g_(*t*) and *r*(*t*) depend ambiguously on the overpotential. On the one hand, a higher overpotential (i.e., a lower concentration of depositing ions near the nucleus surface) promotes a higher growth rate at the initial stages, when the influence of neighboring nuclei is small. On the other hand, a higher number density of nuclei at higher overpotentials enhances competition due to the overlap of diffusion fields and leads to a noticeable decrease in the growth rate. The combination of these factors contributes to the formation of the largest nuclei in the selected system at η = 40 mV due to the highest growth rate. As the overpotential increases, the *c*_sr_ value and its effect on the growth kinetics decrease. At sufficiently high overpotentials (at η ≥ 80 mV in this case), the growth rate depends mainly on the number density of nuclei, i.e., the growth current and the nucleus size decrease as *N*_0_ increases.

Comparison with curves for a single (independent) nucleus demonstrates a decrease in the growth current and the size of nuclei due to the mutual influence of neighbors (see Figure 4b,c). Figure 4d shows the logarithmic dependences needed to determine the growth exponent from slopes of two straight-line sections. It is known that the growth exponent can vary from a value of 1/2, corresponding to the stationary growth of the independent nucleus, to a value close to the theoretical limit of 1/6, corresponding to growth due to purely planar diffusion [19,26,31,57]. The simulation shows that the slopes *x*_1_ and *x*_2_ depend on the overpotential and the number density of nuclei. At high η and *N*_0_ (dark blue curve in Figure 4d), the value of *x*_1_ decreases significantly, which is in good agreement with [19,36], and the value of *x*_2_ ≈ 1/6 can be achieved.

**Figure 5:**
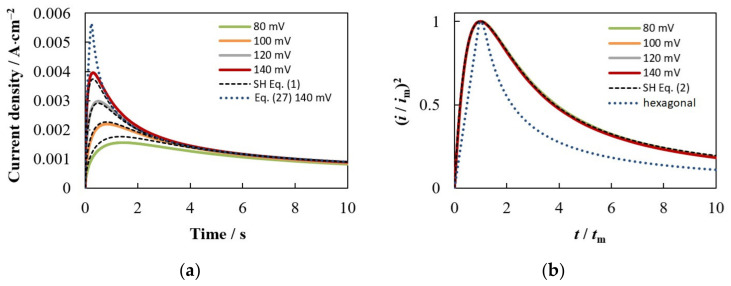
(**a**) Comparison of current density transients from Figure 4a with models based on the concept of planar diffusion zones for random (Equation (1)) and hexagonal (Equation (27)) ensembles; (**b**) Comparison of the same curves in dimensionless form.

Figure 5a demonstrates a comparison of several *i*(*t*) curves from Figure 4a with the SH model and the theoretical dependence derived in [38,39] for instantaneous nucleation followed by diffusion-controlled growth of a hexagonal array using the concept of planar diffusion zones.

(27) $$i(t)=\left\{\begin{array}{l} i_{C}\pi \tilde{k}DN_{0}t,\;t<\gamma^{-1}\\ \sqrt{3}i_{C}\left[\sqrt{\gamma t-1}+\gamma t\left((\pi /6)-\arctan\sqrt{\gamma t-1}\right)\right],\;\gamma^{-1}\leq t\leq {\left(0.75\gamma \right)}^{-1}\\ i_{C},\;t>{\left(0.75\gamma \right)}^{-1} \end{array}\right.,$$

where $i_{C}=zec_{0}{\left(D/\pi t\right)}^{1/2}$ and $\gamma =2\sqrt{3}N_{0}\tilde{k}D$. The maximum of this dependence is unambiguously related to the maximum of the SH model (Equation (3)), namely, *i*_m Equation (27)_ = 1.510 *i*_m SH_ and *t*_m Equation (27)_ = 0.778 *t*_m SH_ ceteris paribus. Therefore, we presented in Figure 5a only one curve calculated by Equation (27) for the highest number density of nuclei.

As can be seen, the simulated dependences for the hexagonal ensembles are quite close to the current density transients calculated by Equation (1) (Figure 5a), and the dimensionless dependences (*i*/*i*_m_)^2^ vs. *t*/*t*_m_ almost coincide with the curve calculated by Equation (2) (Figure 5b). Thus, they agree much better with the SH model designed for random spatial distribution of nuclei than with the model for a hexagonal array [39]. The same trend was observed in Brownian dynamic simulation of the diffusion-controlled growth of hexagonal ensembles in [36].

These discrepancies can be explained as follows. In models based on the concept of planar diffusion zones, the radii of all zones are the same in the case of instantaneous nucleation, *r*_d_ = (*kDt*)^1/2^, and are proportional to the radius of an independent nucleus growing under stationary conditions, *r* = (2*Dc*_0_υ*t*)^1/2^, and the effect of the overpotential is detected only in the *N*_0_ value. This leads to an overestimation of the propagation rate of 2D zones, an increase in the current up to the transition to planar diffusion, and a decrease in the time to reach the maximum, which is especially noticeable when the nuclei are regularly arranged (in a square or hexagonal ensemble). Our model takes into account that the growth rate depends on the number density of nuclei and overpotential (via *c*_sr_) and significantly decreases with time as a result of the overlap of 3D neighboring diffusion fields, which is consistent with the experimental and theoretical results [19,23,26,31,49,57]. The change in relative position of the simulated and SH current density transients (cf. solid and dashed lines in Figure 5a) is explained by the decrease in *c*_sr_ as the overpotential increases. Our simulations show that at high overpotential, *i*_m hex_ will always be higher than *i*_m SH_, but not by a factor of 1.5: *i*_m hex_ = 1.12 *i*_m SH_ and *t*_m Equation (27)_ = 1.01 *t*_m SH_ in the limit *c*_sr_ = 0.

### 3.2. Random Distribution of Nuclei

**Figure 6:**
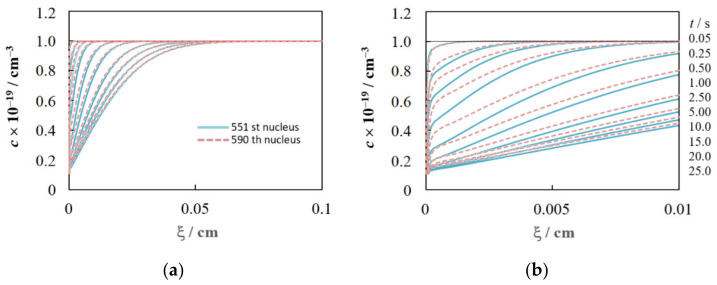
Concentration profiles of 551st (*S*_V_ = 7.06 × 10^−7^ cm^2^, *R*_V_ = 2.95 × 10^−4^ cm) and 590th (*S*_V_ = 8.77 × 10^−7^ cm^2^, *R*_V_ = 4.89 × 10^−5^ cm) nuclei (see Figure 2a) at the times indicated on the right; (**a**,**b**) differ only in ξ scale. η = 100 mV. Values for other parameters are specified in Section 2.3.

In this case, *c*(ξ,*t*) dependences are individual for each nucleus and depend not only on *c*_0_, *c*_sr_(η), and *N*_0_, but also on the location relative to the neighbors, i.e., from the Voronoi cell area, *S*_v_ (cm^2^), and the distance to the nearest side of the cell, *R*_v_ (cm). Figure 6 demonstrates this fact for the concentration profiles of two nuclei from the random ensemble shown in Figure 2a.

**Figure 7:**
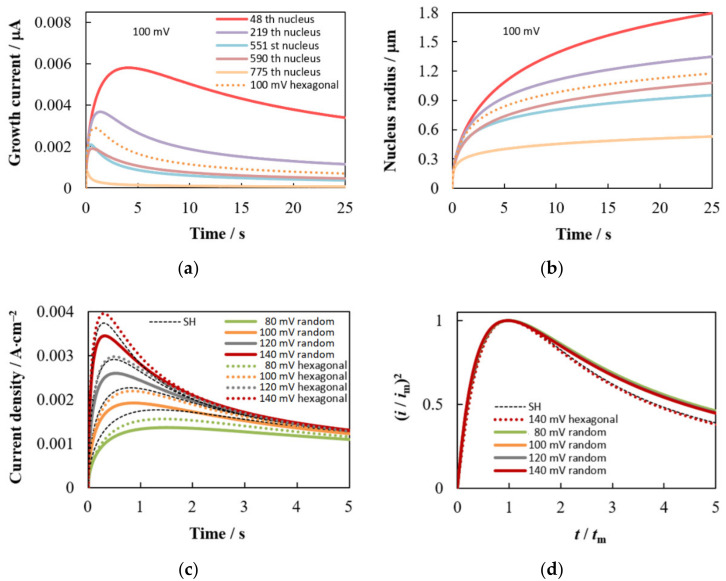
(**a**) Time dependences of the growth current, (**b**) time dependences of the nucleus radius for five nuclei highlighted in Figure 2a; (**c**) Comparison of the total current density transients for random and hexagonal ensembles with the SH model at the same number density of nuclei; (**d**) Dimensionless dependencies for data of (**c**). The same designations of colored lines are used in (**a**,**b**). Parameter values are given in Section 2.3 and Table 1.

Differences in the conditions of mass transfer to each nucleus, associated with the inhomogeneity of the spatial distribution, lead to a difference in the growth rates of the nuclei and, as a result, to a dispersion of their sizes, despite the same initial radius (Figure 7a,b). The nucleus radius increment mainly depends on the Voronoi cell area and, to a lesser extent, on the distance to the nearest neighbor. The smaller *S*_V_ and *R*_V_ result in the smaller nucleus radius and the greater deviation from the growth law of the independent nucleus at large *t*. For example, at η = 100 mV, the growth exponent *x*_2_ = 0.2544 for the largest 48th nucleus (*S*_V_ = 6.13 × 10^−6^ cm^2^, *R*_V_ = 3.34 × 10^−4^ cm), while *x*_2_ = 0.1752 for the smallest 775th one (*S*_V_ = 1.17 × 10^−7^ cm^2^, *R*_V_ = 9.23 × 10^−5^ cm). The indicated values of *x*_1_ and *x*_2_ are in good agreement with the experimental data [19] and the results of Brownian dynamics simulation [36].

An increase in the number density of nuclei contributes to a decrease in the growth current and nucleus radius, but an increase in the total current density (Figure 7c). This figure also shows that the maximum value of the current density for the random ensemble *i*_m rand_ is always lower than for the hexagonal one at the same number density of nuclei. In the limit *c*_sr_ = 0, *i*_m hex_ = 1.15 *i*_m rand_ and *t*_m hex_ = 0.97 *t*_m rand_. In addition, *i*_m rand_ is always lower than *i*_m SH_ (*i*_m rand_ = 0.98 *i*_m SH_ and *t*_m rand_ = 1.04 *t*_m SH_ at *c*_sr_ = 0) because the moment of reaching the maximum is individual for each nucleus. For this reason, there is the deviation of the dimensionless dependences from the SH model (Figure 7d), similar to that often observed in experiments. Qualitatively similar results were obtained at low concentrations of depositing ions in other simulations [29,31,33,36].

Note that the SH model was developed for high overpotentials, when *c*_sr_ can be considered equal to zero and the effect of the charge transfer stage can be safely neglected. However, the SH model is often used in practice at moderate overpotentials. Simulation shows that this can lead to serious errors in determining *N*_0_ and *D* due to the above difference in the values of (*i*_m_, *t*_m_)_rand_ compared to (*i*_m_, *t*_m_)_SH_, even if the growth is indeed diffusion controlled. For example, the error in the *D* value calculated from the product *i*_m_^2^*t*_m_ will be more than 22% at η = 100 mV in our case (*i*_m rand_ = 0.85 *i*_m SH_ and *t*_m rand_ = 1.08 *t*_m SH_ for the orange curve in Figure 7c). At *T* = 298 K, the error will be smaller and amount to about 5% ceteris paribus.

**Figure 8:**
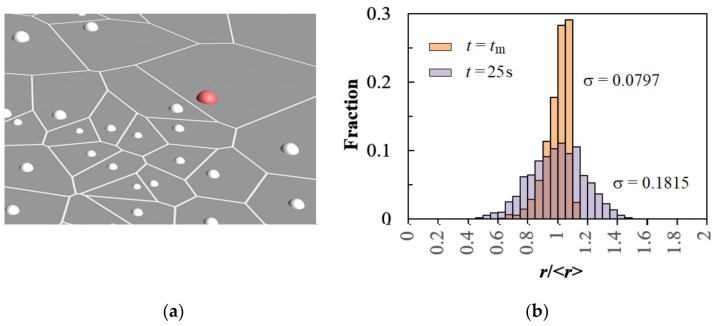
(**a**) Simulated 3D image of the electrode area with nuclei after diffusion-controlled growth of a random ensemble (see Figure 2a) for 25 s at η = 100 mV. The largest 48th nucleus is highlighted in red. (**b**) Nucleus radius distribution for the 1000 nuclei random ensemble. *N*_0_ = 7.55 × 10^5^ cm^−2^, *t*_m_ = 0.875 s.

The magnified fragment of the substrate with nuclei at *t* = 25 s and the size distribution are shown in Figure 8. As can be seen, the radii of nuclei can differ significantly from the average radius <*r*>, the fraction of the largest nuclei decreases, and the dispersion increases during electrodeposition (see also Video S1 in Supplementary Materials). The characteristic asymmetry of the distribution (a tail on the low *r*/<*r*> side) and a 5–10% deviation of the size of nuclei from the mean value (at *t* ≤ 5*t*_m_) were also observed in the simulation of instantaneous nucleation followed by diffusion-controlled growth in [29,31,33,36,37]. The change in the shape of histograms with an increase in the process duration is similar to that mathematically justified in [31].

In conclusion, we emphasize that our results are in good agreement not only with the results of numerical simulation performed using other approaches, but also with experimental data (see, for example, [19,49]). In particular, the correctness of our approach is confirmed by the experimental facts obtained by Lemineur et al. [49] during the in situ observation of the electrodeposition process of silver nanoparticles from the 0.1 M NaNO_3_-100 µM AgNO_3_ solution, registration of individual transients for nanoparticles, and spatial distribution monitoring by Voronoi tessellations using backside absorbing layer microscopy (BALM). Namely, (i) the growth kinetics of an individual nanoparticle is related to the Voronoi cell area and (ii) the summation of individual nanoparticle transients makes it possible to obtain a total chronoamperogram identical to that recorded by the conventional method.

It is important that the proposed model in its present form is suitable for use with any time dependence of the overpotential. For example, this model can be used to calculate cyclic voltammograms. Subsequently, we also plan to apply the proposed approach to simulate more complex cases, including mixed growth control and progressive nucleation.

## 4. Conclusions

The numerical model is proposed for diffusion-controlled growth following the instantaneous nucleation, which considers the spatial distribution of nuclei and the decrease in the growth rate due to the collision of 3D diffusion fields. The model allows us to calculate the fluxes of depositing ions to each nucleus, taking into account the influence of nearest neighbors, and to study the evolution of the system during electrodeposition. The proposed approach provides not only the simulation of the total current density transient, but also comprehensive information about the concentration profiles, individual current transients, and time dependences of the nucleus radius.

Comparison of simulation results for hexagonal ensembles with models using the concept of planar diffusion zones showed that the total current density transients agree much better with the SH model for instantaneous nucleation and diffusion-controlled growth of random ensembles (especially in dimensionless form) than with the model [39] for hexagonal arrays. The reason is apparently the overestimated growth rate of identical 2D zones used in these models. According to our estimate, the maximum current density of the hexagonal ensemble exceeds that in the SH model by no more than 1.12 times at the same number density of nuclei. Calculations show that the growth exponent in the case of the growth of hexagonal ensembles can vary from 1/2 to 1/6 depending on the electrodeposition time, number density of nuclei, and overpotential.

In the case of a random spatial distribution of nuclei, the conditions for mass transfer to each nucleus depend on the Voronoi cell area and, not so obviously, on the distance to the nearest neighbor. For this reason, the growth exponents are individual for each nucleus, and a dispersion of the sizes of the nuclei arises, despite the identical initial radius. The size dispersion increases as the electrodeposition duration increases. An increase in the number density of nuclei contributes to a decrease in the growth current and nucleus radius, but an increase in the total current density. The maximum current density value for the random ensemble is always lower than for the hexagonal one and SH model at the same number density of nuclei (*i*_m hex_ = 1.15 *i*_m rand_, *t*_m hex_ = 0.97 *t*_m rand_ and *i*_m rand_ = 0.98 *i*_m SH_, *t*_m rand_ = 1.04 *t*_m SH_ at *c*_sr_ = 0). The influence of the concentration of depositing ions near the nucleus surface cannot be neglected at moderate overpotentials, so the use of Equation (3) can introduce an error into the values of the diffusion coefficient of depositing ions and the number density of nuclei. Detected deviation of the dimensionless dependences *i*/*i*_m_ vs. *t*/*t*_m_ from the SH model is similar to that often observed in experiments and is associated with different growth rates of nuclei.

Comparison of the simulation results with reliable data from the literature confirms the adequacy of the proposed approach and the prospects of its use for the analysis of electrochemical phase formation in more complex cases.

## Figures and Tables

**Table 1:** Values of the overpotential, number density of nuclei, and the half-distance between the nearest neighbors (for hexagonal ensembles).

η, mV	^1^*N*_0_, cm^−2^	*R*, cm
10	7.96 × 10^4^	2.00 × 10^−3^
20	1.02 × 10^5^	1.76 × 10^−3^
40	1.69 × 10^5^	1.37 × 10^−3^
80	4.58 × 10^5^	8.34 × 10^−4^
100	7.55 × 10^5^	6.49 × 10^−4^
120	1.25 × 10^6^	5.06 × 10^−4^
140	2.05 × 10^6^	3.94 × 10^−4^

^1^ *N*_0_(η) = 6.2 × 10^4^ exp (0.025η) in this paper.

## Data Availability

The data presented in this study are available on request from the corresponding author.

## Supplementary Materials

The following supporting information can be downloaded at: https://www.mdpi.com/article/10.3390/ma15103603/s1, Video S1: Growth of nuclei located near the smallest 775th nucleus.
